# Supplementary material for: Proactively location-based suppression elicited by statistical learning
Source: PLoS One. 2020 Jun 1;15(6):e0233544. doi: 10.1371/journal.pone.0233544 (PMC7263585; doi:10.1371/journal.pone.0233544)
Supplement: S1 Appendix — (DOCX) [file pone.0233544.s001.docx]

**Appendix**

This appendix includes the analysis of the training *search-only* trials. Trials on which the response times (RTs) were slower than 1500 ms or quicker than 200 ms (5.4%) were removed from analysis.

**Attentional capture effect**

Mean RTs and mean error rates are presented in Fig A1A. With *distractor condition* (high-probability location, low-probability location, and no-distractor) as a factor, a repeated measures ANOVA on mean RTs showed a main effect, *F*(2, 30) = 90.17, *p* < .001, *η_p_^2^* = .86. Subsequent planned comparisons showed that there were significant attentional capture effects when the distractor singleton was presented at the high-probability location, *t*(15) = 8.96, *p* < .001, *cohen’s d* = 0.64, and when it was presented at the low-probability location, *t*(15) = 10.65, *p* < .001, *cohen’s d* = 1.3. Importantly, consistent with Wang and Theeuwes [1, 14-15], the difference between high- and low-probability locations was reliable, *t*(15) = 7.38, *p* < .001, *cohen’s d* = 0.69, suggesting that the attentional capture effect was attenuated for trials in which the distractor singleton appeared at the high-probability location.

We also observed a significant main effect on error rates, *F*(2, 30) = 5.95, *p* = .007, *η_p_^2^* = .28. Subsequent comparisons showed that participants made more mistakes, compared to no-distractor condition, when the distractor singleton was presented at the low-probability location, *t*(15) = 3.42, *p* = .004, *cohen’s d* = 0.66, but not when it was presented at the high-probability location , *t*(15) = 0.21, *p* = .837, *cohen’s d* = 0.05, BF01 = 3.84. Again, it was significantly different between high- and low-probability locations, *t*(15) = 2.63, *p* = .019, *cohen’s d* = 0.68.

**Target selection**

Mean RTs and mean error rates are presented in Fig A1B. To further examine whether the efficient for target selection was reduced or not in the distractor singleton absent condition, paired-wise t-test showed that the selection was less efficient when the target was presented at the high-probability location compared to when it was presented at the low-probability location, *t*(15) = 2.87, *p* = .012, *cohen’s d* = 0.37. There was no effect on error rates, *t*(15) = 0.93, *p* = .369, *cohen’s d* = 0.28, BF01 =2.7.

**Fig A1.** The mean response times and mean error rates in different distractor conditions (A) and in the distractor singleton absent condition (B)
